# A comparison of various feature extraction and machine learning methods for antimicrobial resistance prediction in *streptococcus pneumoniae*

**DOI:** 10.3389/frabi.2023.1126468

**Published:** 2023-03-24

**Authors:** Deniz Ece Kaya, Ege Ülgen, Ayşe Sesin Kocagöz, Osman Uğur Sezerman

**Affiliations:** ^1^ Department of Biostatistics and Medical Informatics, School of Medicine, Acibadem Mehmet Ali Aydinlar University, Istanbul, Türkiye; ^2^ Department of Infectious Diseases, School of Medicine, Acibadem Mehmet Ali Aydinlar University, Istanbul, Türkiye

**Keywords:** AMR, machine learning, streptococcus pneumonaie, SNP, kmer, whole genome sequencing (WGS)

## Abstract

Streptococcus pneumoniae is one of the major concerns of clinicians and one of the global public health problems. This pathogen is associated with high morbidity and mortality rates and antimicrobial resistance (AMR). In the last few years, reduced genome sequencing costs have made it possible to explore more of the drug resistance of S. pneumoniae, and machine learning (ML) has become a popular tool for understanding, diagnosing, treating, and predicting these phenotypes. Nucleotide k-mers, amino acid k-mers, single nucleotide polymorphisms (SNPs), and combinations of these features have rich genetic information in whole-genome sequencing. This study compares different ML models for predicting AMR phenotype for S. pneumoniae. We compared nucleotide k-mers, amino acid k-mers, SNPs, and their combinations to predict AMR in S. pneumoniae for three antibiotics: Penicillin, Erythromycin, and Tetracycline. 980 pneumococcal strains were downloaded from the European Nucleotide Archive (ENA). Furthermore, we used and compared several machine learning methods to train the models, including random forests, support vector machines, stochastic gradient boosting, and extreme gradient boosting. In this study, we found that key features of the AMR prediction model setup and the choice of machine learning method affected the results. The approach can be applied here to further studies to improve AMR prediction accuracy and efficiency.

## 1 Introduction

Antimicrobial resistance (AMR) has caused a significant increase in morbidity and mortality rate in infectious diseases all over the world. According to World Health Organization (WHO), AMR is one of the top 10 global public health threats humanity faces. The global death rate from infectious diseases is projected to rise to 10 million per year by 2050 (World Health Organization, 2019; World Health Organization, 2022a). Many commonly used antibiotics have become ineffective due to rapidly increasing antimicrobial resistance in pathogens (Blair et al., 2015). In recent years, the development of new antimicrobial compounds has not been as rapid as the spread of resistance (Henriques-Normark and Tuomanen, 2013; Michael et al., 2014; Christaki et al., 2019), and this raises global health concerns. Rapid antibiotic susceptibility tests (AST) can guide the use of antibiotics and reduce drug-resistant strains. Currently, classical phenotypic AST methods, based on culturing target pathogens, are the gold standard. However, these methods take a few days to result and delay urgent treatment decisions. This delay also contributes to the spread of drug resistance (AMR Review, 2015). Molecular approaches have significantly improved over the years and play a critical role in the fight against antimicrobial resistance (Inouye et al., 2014). Due to the rapid development of sequencing technology and the decreasing cost, whole genome sequencing (WGS) or direct metagenomic sequencing of clinical materials has been proposed as the next-generation genotypic AST (Dunne et al., 2017; Zhang et al., 2019). In the face of growing AMR threats, it is increasingly vital to develop methods for interpreting minimum inhibitor concentrations (MICs) tests (Michael et al., 2020). Epidemiological cutoff values are set by the European Committee on Antimicrobial Susceptibility Testing (EUCAST) (ESCMID - European Society of Clinical Microbiology and Infectious Diseases, 2008) and by the Clinical and Laboratory Standards Institute (CLSI) for its epidemiological cutoff values (CLSI guidelines, 2022). Clinical breakpoints are another popular method of categorization. As a result of this process, MIC values are categorized according to different clinical outcomes (Michael et al., 2020). According to CLSI, these classes are “resistant” (R), “susceptible” (S), and “intermediate” (I) (CLSI guidelines, 2022).

As discussed above, due to reduced genome sequencing costs, detecting AMR phenotypes directly from sequence data has become a preferred method. In the last few years, the use of machine learning (ML) for understanding, diagnosing, treating, and predicting AMR phenotypes has aroused interest in the literature, and it has been shown in publications (Yang et al., 2017; Nguyen et al., 2018; Deelder et al., 2019; Nguyen et al., 2019; Khaledi et al., 2020; Wang et al., 2022) that for many bacterial species. Antimicrobial resistance can be predicted quite accurately based on the genome sequence. ML techniques applied to WGS can accurately predict MIC results. However, some MIC data are only shared as classes, while the remaining are shared as concentration, which may cause discrepancies while training ML models.

AMR has been extensively studied *via* ML in various microorganisms, including *Mycobacterium tuberculosis* (Davis et al., 2016; Drouin et al., 2016; Yang et al., 2017; Deelder et al., 2019; Aytan-Aktug et al., 2020; Wang et al., 2022), *Escherichia col*i (Moradigaravand et al., 2018; Pataki et al., 2020; Aytan-Aktug et al., 2020), *Salmonella enterica* (Aytan-Aktug et al., 2020), nontyphoidal Salmonella (Nguyen et al., 2019), *Staphylococcus aureus* (Davis et al., 2016; Aytan-Aktug et al., 2020; ValizadehAslani et al., 2020), *Acinetobacter baumannii* (Davis et al., 2016), *Streptococcus pneumoniae* (Davis et al., 2016; Drouin et al., 2016; Li et al., 2016; Li et al., 2017; Zhang et al., 2019), *Clostridium difficile* (Drouin et al., 2016), *Pseudomonas aeruginosa* (Drouin et al., 2016; Khaledi et al., 2020), *Actinobacillus pleuropneumoniae* (Liu et al., 2020), *Elizabethkingia* (Naidenov et al., 2019), *Klebsiella pneumoniae* (Nguyen et al., 2018; ValizadehAslani et al., 2020), *Campylobacter jejuni* (ValizadehAslani et al., 2020), and *Neisseria gonorrhoeae* (Eyre et al., 2017; ValizadehAslani et al., 2020). While setting up ML models, k-mer counts of various lengths (8-mers to 11-mers (ValizadehAslani et al., 2020), 10-mers (Nguyen et al., 2018), 15-mers (Nguyen et al., 2019), 31-mers (Davis et al., 2016; Drouin et al., 2016)), AMR genes (Her and Wu, 2018), SNPs (Yang et al., 2017; Deelder et al., 2019; Shi et al., 2019), or a combination of these (Moradigaravand et al., 2018; Naidenov et al., 2019; Khaledi et al., 2020) have been successfully used as features. In a recently published study by ValizadehAslani et al. (2020), amino acid k-mers were also utilized as features and yielded successful results.

*Streptococcus pneumoniae* is known to be one of the bacteria with the most common AMR problem (van der Poll et al., 2009). S. *pneumoniae* is a gram-positive human pathogen that is the primary cause of respiratory tract infection and diseases such as pneumonia and meningitis. This bacterium is also found in the nasopharyngeal flora in childhood and often causes invasive infectious diseases such as acute otitis media and sinusitis (Henriques-Normark and Tuomanen, 2013). According to the WHO, diseases caused by Streptococcus pneumoniae are an important public health problem worldwide. It is estimated that about one million children die yearly from pneumococcal disease (World Health Organization, 2022b-2).

Many pneumococcal isolates are resistant to common antibacterial drugs like fluoroquinolones, macrolides, and β- lactams (Sader et al., 2019). The main targets of penicillin are penicillin-binding proteins (PBPs). For many years, penicillin has been the primary choice for treating S.pneumoniae-associated infections (Zapun et al., 2008). β-lactams bind to enzymes essential for bacterial cell wall synthesis and reducing peptidoglycan synthesis. (Zapun et al., 2008). The main resistance mechanism to resist β-lactams is mutating PBPs to reduce their affinity to antibiotics (Poole, 2004). During the same time as penicillin resistance spread, macrolide-resistant pneumococci also increased. Moreover, the removal of the antimicrobial from the cell by the acquisition of *mef* and *erm* genes and modification of the target site are the two main mechanisms of macrolide-like erythromycin resistance in S. pneumoniae (Cornick and Bentley, 2012). Tetracyclines inhibit the growth of bacteria by binding to the 30S subunit of the bacterial ribosome. Pneumococcal resistance to tetracycline occurs *via* ribosomal protection (tet(O) and tet(M) genes) (Montanari et al., 2003).

This study compares different ML models for predicting AMR phenotype for S. pneumoniae. We compared nucleotide k-mers, amino acid k-mers, and SNPs to predict AMR in S. pneumoniae for three antibiotics: Penicillin, Erythromycin, and Tetracycline. Further, we attempted to use and compare various ML methods: random forest (RF), support vector machine (SVM), stochastic gradient boosting (GBM), and extreme gradient boosting (XGBoost) to train the models. We discuss the strengths and limitations of feature and ML model selection for MIC prediction. We observed and concluded that the choice of features and the selection of the ML model affects the performance of prediction (as measured by F1 score and accuracy) differently for each antibiotic.

## 2 Methods

### 2.1 Overview

**Figure 1:**
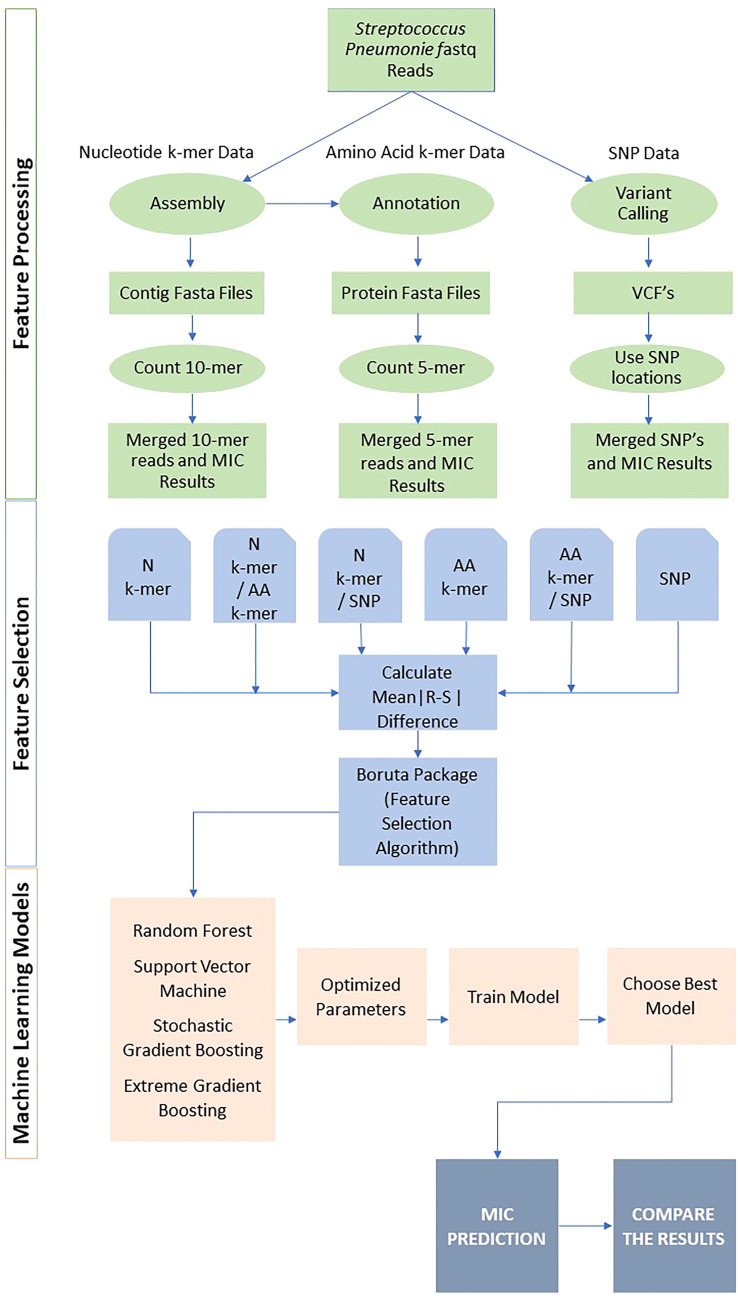
Overall pipeline for all feature extraction and classification approaches.

The overview of the study is presented in **Figure 1**. Following feature generation, feature selection, and various ML models were trained to predict the MIC class for each antibiotic. The performances were evaluated using several metrics, and the results were compared. The details of data, feature generation, feature selection, model training, and evaluation are described in the following subsections. All analyses were performed in R version 4.0.2 (http://www.R-project.org). The R scripts utilized in this study are available on GitHub at https://github.com/denizecek/AMRprediction.

### 2.2 Datasets and pre-processing

S. pneumoniae metagenomic sequences and the related MIC class information of the antibiotics penicillin, erythromycin, and tetracycline were included in our study. We used four publicly available datasets: 980 pneumococcal strains were downloaded from the European Nucleotide Archive (ENA) (http://www.ebi.ac.uk/ena/) with the project accession code in PRJEB2632 (Croucher et al., 2013; Croucher et al., 2015), PRJNA34791 (Demczuk et al., 2017), PRJEB3084 (Gladstone et al., 2019), PRJEB2255 (Croucher et al., 2014; Croucher et al., 2015). The MIC class information was downloaded from the PATRIC database (Davis et al., 2020) and PubMLST (Jolley et al., 2018), and the “Resistant” and “Susceptible” classes were matched to the genome data. For each antibiotic, we discarded the “Intermediate” class because these were underrepresented. **Table 1** presents the sample number of the four datasets, and **Table S1** (**Supplementary 1**) contains detailed sample information.

**Table 1:** Datasets and the corresponding numbers of samples per MIC class for the three antibiotics.

Antibiotic	DS 1 - PRJEB2632		DS 2 – PRJEB3084		DS 3 – PRJNA347910		DS 4 - PRJEB2255		Total
	S	R	S	R	S	R	S	R	
Penicillin	453	68	11	49	2	109	128	49	869
Erythromycin	470	78	18	41	25	132	17	114	895
Tetracycline	287	36	35	19	3	131	11	121	643

### 2.3 Feature generation and selection

#### 2.3.1 Nucleotide K-mers

SPAdes (Bankevich et al., 2012) in the PATRIC assembly service (Davis et al., 2020) was used for genome assembly. Contigs with less than 5-fold coverage and lengths less than 500 bp were removed. The contigs were divided into 10-mers, and the frequencies of these 10-mers were obtained using the R “kmer” package (Wilkinson, 2018). For the AMR classification task, the k-mer counts were used as one set of features, and antibiotic MIC classes were used as labels.

In this work, we chose to use a 10-mers instead of a longer k-mer length to reduce the size of the resulting k-mer matrix. Longer k-mers were not selected because of memory limitations, and we did not utilize shorter k-mers due to lower initial accuracy. Next, k-mer counts were converted to depict the presence “1” or absence “0” of each k-mer in each genome.

The dataset was very large, and fitting ML models using this data might have caused significant challenges, including high computational cost and processing time. Moreover, it is known that ML models trained on large datasets (i.e., large sets of features) tend to have poorer performance compared to using an optimal set of features (Yu and Liu, 2004; Pudjihartono et al., 2022). Since the total number of 10-kmers is 1,048,578 in our dataset, the absolute mean difference between resistant and susceptible samples of each feature was used as the first feature selection step. For every feature, we calculated the mean of the resistance and susceptible samples, features with an absolute mean difference of at least 0.3 were selected for penicillin and erythromycin and 0.4 were selected for tetracycline. The main reason we choose 0.3 as the threshold is to reduce the number of features below 10000. When this cutoff value was set to 0.2, 11149 features remained for penicillin, and when it was set to 0.3, 2099 features remained. When 0.4 was selected, we had 141 features remaining. Final number of features before the next feature selection step for penicillin, erythromycin and tetracycline are 1591, 2099 and 3376.

#### 2.3.2 Single nucleotide polymorphisms

We used single nucleotide polymorphisms (SNPs) as another set of features for ML model training. Our reference genome for SNP calling was *S. pneumoniae* TIGR4. For variant calling, BWA-mem (Li, 2013) and SAMtools (Li et al., 2009) were used *via* the PATRIC variant calling service (Davis et al., 2020). Bcftools (Li, 2011) was used for filtering variants with DP > 20 and qual > 50 parameters. A total of 221,304 SNPs were obtained. SNP positions (compared to the reference genome) were the columns of the resulting matrix, and the samples were rows. A sample with an SNP at a given site was shown as 1, and those without any SNPs were shown as 0. Compared to the 10-mer features, the number of SNP features was much lower (221,304); hence the absolute mean difference cutoff value was also decreased. The absolute mean difference between resistant and susceptible samples was calculated for each SNP and filtered at least 0.2 for all three antibiotics. The features with an absolute mean difference lower than this cutoff were removed. With this first step of feature selection, for penicillin, 4,954 features remained; for erythromycin, 8,844 features remained and for tetracycline, 6,695 features remained.

#### 2.3.3 Amino acid K-mers

An amino acid k-mer model for predicting MIC classes for the three antibiotics was built following the method previously described by ValizadehAslani et al. (2020). To provide annotation of genomic features, the Genome Annotation Service in PATRIC (Davis et al., 2016), which uses the RAST toolkit (RASTtk) (Brettin et al., 2015), was utilized. Protein FASTA sequences were downloaded from the PATRIC database. For counting the amino acid k-mers, we used the “kmer” R package (Wilkinson, 2018). The Dayhoff-6 alphabet (Edgar, 2004) was used to minimize computation time when counting longer k-mers. 5-mers of the amino acid were counted for the genome of each strain. We did not use shorter amino acid k-mers due to lower initial accuracy, and longer k-mers were not chosen because of memory limitations. Since the total number of features was less than 10,000, the pre-elimination used in other feature extraction methods (absolute mean difference between MIC classes) was not used here.

#### 2.3.4 Combinations of features

10-mer nucleotides, 5-mer amino acid content, and SNP features were combined as binary combinations and tested as another feature extraction method. Sections 2.3.1, 2.3.2, and 2.3.3 were used for feature selection, and the optimal features were combined.

#### 2.3.5 Boruta

Feature selection algorithm Boruta, implemented as an R package, was used for the second and final feature selection step. Boruta is an ML algorithm used in feature selection (Kursa and Rudnicki, 2010). It is a wrapper feature selection method built around the Random Forest classification algorithm. The algorithm adds randomness to the data set by creating a shuffled copy of all features. These features are called “Shadow Features”. The shadow features and original features are then merged, and the algorithm builds a random forest classifier, which determines each feature’s importance using Z-score and mean decreased accuracy. Boruta then checks whether an original feature has higher importance than the shadow features. At each iteration, significant features are kept, and unimportant ones are constantly removed. This iteration repeats until all features are confirmed or rejected (Kursa and Rudnicki, 2010). In this study, the important features (**Table 2**) were chosen using the default settings of Boruta.

**Table 2:** Boruta Results and number of Important (Imp), Unimportant (Unimp), and Tentative (Ten) features.

Antibiotic	Nucleotide 10-mer Boruta Results			Amino Acid 5-mer Boruta Results			SNP Boruta Results		
	Imp	Unimp	Ten	Imp	Unimp	Ten	Imp	Unimp	Ten
Penicillin	138	4690	126	162	8394	288	25	6628	42
Erythromycin	153	4633	168	45	8660	139	124	6394	177
Tetracycline	108	4642	204	18	8736	90	106	6432	157

#### 2.3.6 Statistical Analysis

To understand the importance of nucleotide 10-mers, top ten features were determined for each model. The union of these most successful features consisting of nucleotide 10-mers has been prepared. Hypergeometric distribution test was used to determine whether these kmers were over-represented in AMR genes. For penicillin samples, pbp2B, pbp2x, pbp1a genes were downloaded from NCBI, the contigs were divided into 10-mers, and hypergeometric distribution was calculated for each antibiotic.

Ermb, mefE, mefA were used for erythromysin nucleotide 10-mers and tetS and tetM were for tetracycline.

### 2.4 Machine learning models for AMR classification

As samples to generate the ML models, as discussed above, since the small number of intermediate samples created an imbalance, we only included isolates categorized as either “resistant” or “susceptible” for each antibiotic. As features, we separately utilized the presence/absence of SNPs, nucleotide 10-mers, and amino acid 5-mers. Pairs of combinations of these features were analyzed in the same manner. An experiment of tenfold cross validation was used to evaluate the model’s stability and accuracy and was built according to the methodology previously described by Davis et al. (2020). For each drug, we randomly assigned isolates to a training set comprising 80% of the resistant and susceptible isolates, respectively. The remaining 20% were divided equally into a test set and a validation set. Parameters of ML models were optimized on the validation set, and their accuracy was assessed in cross-validation, while the test set was used to obtain another independent performance estimate.

The accuracy and sensitivity of the ML models generated by this study were evaluated by 10-fold cross-validation. The data were divided into training and test sets as 8:2. The matrix is divided into ten equal parts by cross-validation, with an equal number of antibiotic-MIC combinations in each part. One part is used for testing, one for validation, and eight for training. Each model used the validation set to avoid overfitting. 10-fold cross-validation was performed in the hyperparameter tuning stage. Optimal combinations of hyper-parameters were selected for each fold based on the mean squared error of validation. Ten sets of hyper-parameters were generated from the tests, one for each fold. Different ML algorithms were compared based on accuracy, F1 score, and Cohen’s (unweighted) Kappa statistic averaged across the resampling results.

To detect penicillin, erythromycin, and tetracycline resistance *Streptococcus pneumonia*, we trained random forest (RF), support vector machine (SVM), stochastic gradient boosting (GBM), and extreme gradient boosting (XGBoost) classifiers. Three different models were tested for RF. The model with the default for each parameter, random search, and grid search was performed. For the SVM classifier, SVM with linear kernel, polynomial and radial kernel functions were tested. For the GBM we tried the tuning parameters. For XGBoost models, we used a grid search to tune our important hyperparameters. The models with the highest F1 score among all created models were compared. The optimal parameters for each ML approach are presented in **Table S2**.

Notably, the relative contribution of the different information sources to the susceptibility and resistance sensitivity strongly depended on the antibiotic. To assess the effect of the classification technique, we compared the performance of different classifiers. The 980-genome model contained data from all antibiotics and MICs, making feature extraction challenging to determine which k-mers contribute to the MIC predictions for each antibiotic. To address this limitation, we modified the protocol by building separate models for each antibiotic. Another reason why we set up a separate model for each antibiotic is that not all samples have MIC information for all three antibiotics. As you can see in **Table 1** we have 869 penicillin samples, however we have 643 tetracycline samples with MIC information. We did not want to reduce the number of samples to train our models with a single large integrated model. We also worried about the computational problems like memory, RAM and training times to performing best classifier for a single large integrated models for all antibiotics. 

## 3 Results

As detailed in Methods, we trained several ML classification methods on features individually and in combination for predicting antibiotic susceptibility or resistance of isolates and evaluated the classifier performances. We calculated the accuracy, sensitivity, specificity, and the F1-score, as an overall performance measure based on a classifier trained on a specific combination and shown in **Tables 3**–**5**. Training and validation sets accuracy and kappa results are presented in **Tables S3**–**S5**.

**Table 3:** Penicillin models performances.

Algorithm	Input	F1-score	Kappa	Accuracy	Sensitivity	Specificity
Random Forest	k-mer	0.848	0.814	0.943	0.849	0.965
Support Vector Machine	k-mer	0.865	0.834	0.950	0.852	0.972
Stochastic Gradient Boosting	k-mer	0.879	0.851	0.955	0.879	0.972
Extreme Gradient Boosting	k-mer	0.866	0.843	0.950	0.853	0.972
Random Forest	AA k-mer	0.900	0.871	0.961	0.882	0.980
Support Vector Machine	AA k-mer	0.899	0.874	0.961	0.861	0.986
Stochastic Gradient Boosting	AA k-mer	0.882	0.854	0.955	0.857	0.979
Extreme Gradient Boosting	AA k-mer	0.886	0.858	0.955	0.838	0.986
Random Forest	SNP	0.786	0.747	0.932	0.957	0.929
Support Vector Machine	SNP	0.772	0.730	0.927	0.917	0.928
Stochastic Gradient Boosting	SNP	0.759	0.712	0.921	0.880	0.928
Extreme Gradient Boosting	SNP	0.786	0.747	0.932	0.957	0.929
Random Forest	SNP/AA k-mer	0.889	0.865	0.961	0.933	0.966
Support Vector Machine	SNP/AA k-mer	0.889	0.865	0.961	0.933	0.966
Stochastic Gradient Boosting	SNP/AA k-mer	0.857	0.826	0.950	0.900	0.959
Extreme Gradient Boosting	SNP/AA k-mer	0.871	0.844	0.955	0.931	0.960
Random Forest	SNP/k-mer	0.831	0.793	0.938	0.844	0.959
Support Vector Machine	SNP/k-mer	0.820	0.782	0.938	0.893	0.946
Stochastic Gradient Boosting	SNP/k-mer	0.852	0.822	0.949	0.929	0.963
Extreme Gradient Boosting	SNP/k-mer	0.867	0.840	0.955	0.953	0.953
Random Forest	AA k-mer/k-mer	0.867	0.840	0.955	0.963	0.953
Support Vector Machine	AA k-mer/k-mer	0.852	0.822	0.950	0.929	0.953
Stochastic Gradient Boosting	AA k-mer/k-mer	0.847	0.818	0.950	0.962	0.947
Extreme Gradient Boosting	AA k-mer/k-mer	0.847	0.818	0.950	0.962	0.947

**Table 4:** Erythromycin models performances.

Algorithm	input	F1-score	Kappa	Accuracy	Sensitivity	Specificity
Random Forest	k-mer	0.961	0.944	0.977	0.961	0.984
Support Vector Machine	k-mer	0.940	0.916	0.965	0.960	0.968
Stochastic Gradient Boosting	k-mer	0.951	0.931	0.971	0.942	0.984
Extreme Gradient Boosting	k-mer	0.971	0.959	0.983	0.962	0.991
Random Forest	AA k-mer	0.952	0.932	0.971	0.926	0.992
Support Vector Machine	AA k-mer	0.952	0.932	0.971	0.926	0.992
Stochastic Gradient Boosting	AA k-mer	0.961	0.944	0.977	0.961	0.983
Extreme Gradient Boosting	AA k-mer	0.961	0.944	0.977	0.961	0.983
Random Forest	SNP	0.813	0.748	0.902	0.925	0.895
Support Vector Machine	SNP	0.821	0.754	0.902	0.886	0.907
Stochastic Gradient Boosting	SNP	0.816	0.744	0.896	0.851	0.913
Extreme Gradient Boosting	SNP	0.792	0.712	0.884	0.844	0.898
Random Forest	SNP/AA k-mer	0.876	0.822	0.925	0.851	0.958
Support Vector Machine	SNP/AA k-mer	0.884	0.835	0.930	0.867	0.958
Stochastic Gradient Boosting	SNP/AA k-mer	0.862	0.805	0.919	0.862	0.942
Extreme Gradient Boosting	SNP/AA k-mer	0.873	0.820	0.924	0.865	0.950
Random Forest	SNP/k-mer	0.944	0.919	0.965	0.894	1.000
Support Vector Machine	SNP/k-mer	0.927	0.893	0.953	0.864	1.000
Stochastic Gradient Boosting	SNP/k-mer	0.914	0.877	0.948	0.889	0.974
Extreme Gradient Boosting	SNP/k-mer	0.884	0.835	0.930	0.867	0.958
Random Forest	AA k-mer/k-mer	0.932	0.903	0.959	0.923	0.975
Support Vector Machine	AA k-mer/k-mer	0.942	0.917	0.965	0.924	0.983
Stochastic Gradient Boosting	AA k-mer/k-mer	0.932	0.903	0.959	0.923	0.975
Extreme Gradient Boosting	AA k-mer/k-mer	0.900	0.859	0.942	0.918	0.951

**Table 5:** Tetracycline models performances.

Algorithm	input	F1-score	Kappa	Accuracy	Sensitivity	Specificity
Random Forest	k-mer	0.906	0.867	0.944	0.850	0.988
Support Vector Machine	k-mer	0.921	0.887	0.952	0.853	1.000
Stochastic Gradient Boosting	k-mer	0.891	0.847	0.937	0.846	0.977
Extreme Gradient Boosting	k-mer	0.891	0.847	0.937	0.846	0.977
Random Forest	AA k-mer	0.933	0.905	0.960	0.875	1.000
Support Vector Machine	AA k-mer	0.931	0.903	0.960	0.894	0.988
Stochastic Gradient Boosting	AA k-mer	0.933	0.905	0.960	0.875	1.000
Extreme Gradient Boosting	AA k-mer	0.917	0.883	0.952	0.891	0.977
Random Forest	SNP	0.869	0.820	0.929	0.882	0.946
Support Vector Machine	SNP	0.849	0.788	0.913	0.815	0.955
Stochastic Gradient Boosting	SNP	0.869	0.820	0.929	0.882	0.946
Extreme Gradient Boosting	SNP	0.873	0.824	0.929	0.861	0.956
Random Forest	SNP/AA k-mer	0.929	0.902	0.960	0.916	0.978
Support Vector Machine	SNP/AA k-mer	0.901	0.863	0.944	0.888	0.967
Stochastic Gradient Boosting	SNP/AA k-mer	0.916	0.883	0.952	0.891	0.997
Extreme Gradient Boosting	SNP/AA k-mer	0.929	0.902	0.960	0.916	0.978
Random Forest	SNP/k-mer	0.906	0.867	0.944	0.850	0.988
Support Vector Machine	SNP/k-mer	0.906	0.867	0.944	0.850	0.988
Stochastic Gradient Boosting	SNP/k-mer	0.876	0.827	0.929	0.842	0.966
Extreme Gradient Boosting	SNP/k-mer	0.891	0.847	0.937	0.846	0.933
Random Forest	AA k-mer/k-mer	0.906	0.867	0.944	0.850	0.988
Support Vector Machine	AA k-mer/k-mer	0.921	0.887	0.952	0.853	1.000
Stochastic Gradient Boosting	AA k-mer/k-mer	0.906	0.867	0.944	0.850	0.988
Extreme Gradient Boosting	AA k-mer/k-mer	0.918	0.885	0.952	0.871	0.988

**Figure 2:**
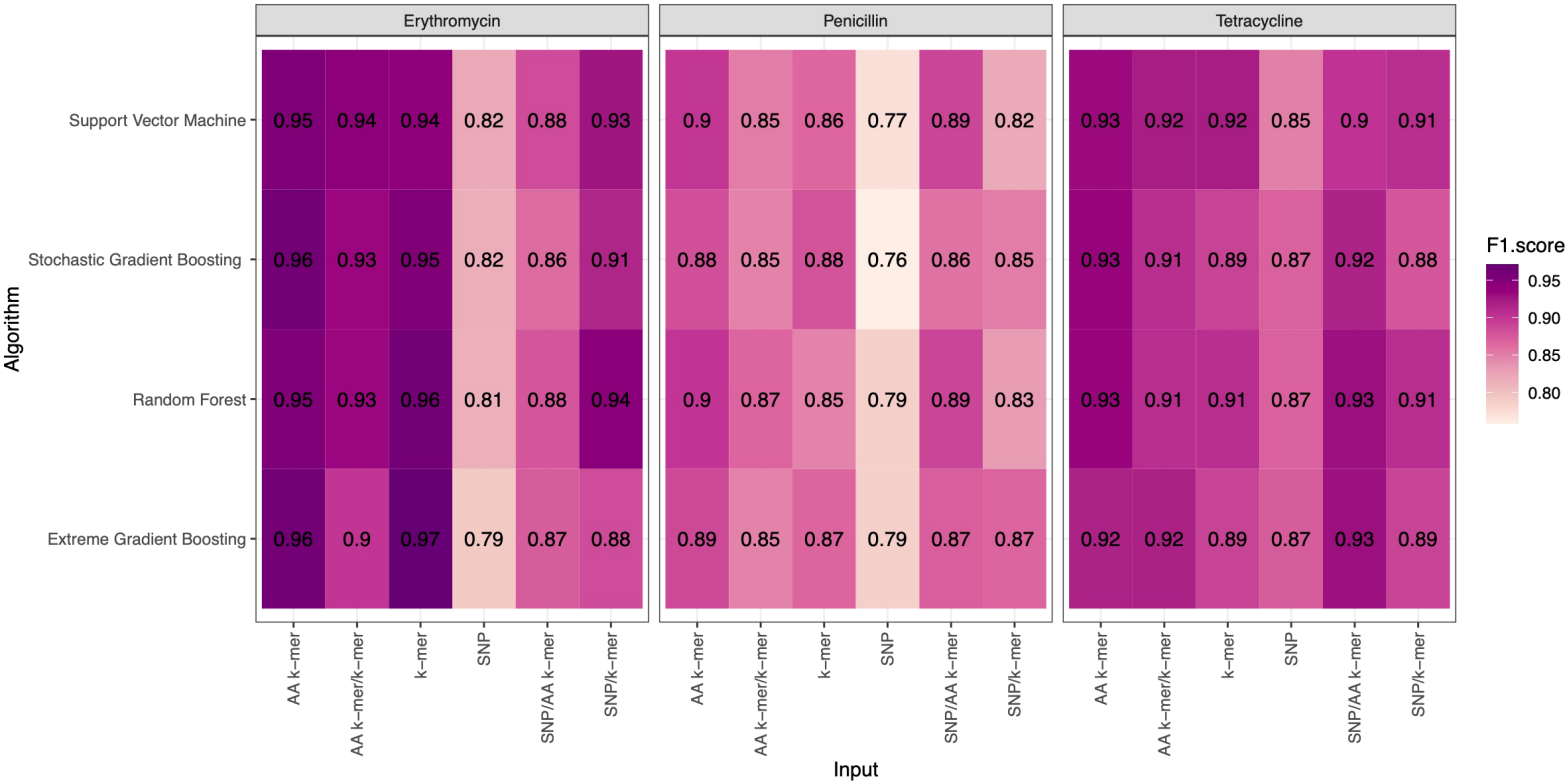
F1 Scores of penicillin, erythromycin, and tetracycline resistance classification models using six different input types and four different ML approaches.

F1 Scores of all penicillin, erythromycin, and tetracycline resistance models using six feature types and 4 ML approaches are presented in **Figure 2**. While the overall performances were adequate, different feature types and ML approaches yielded varying performances per each antibiotic.

Of interest when the distribution of SNPs between resistant and susceptible samples were compared for each antibiotic, it was observed that the number of SNPs in the susceptible samples was significantly higher for all three antibiotics (**Figure S1**).

**Figure 3:**
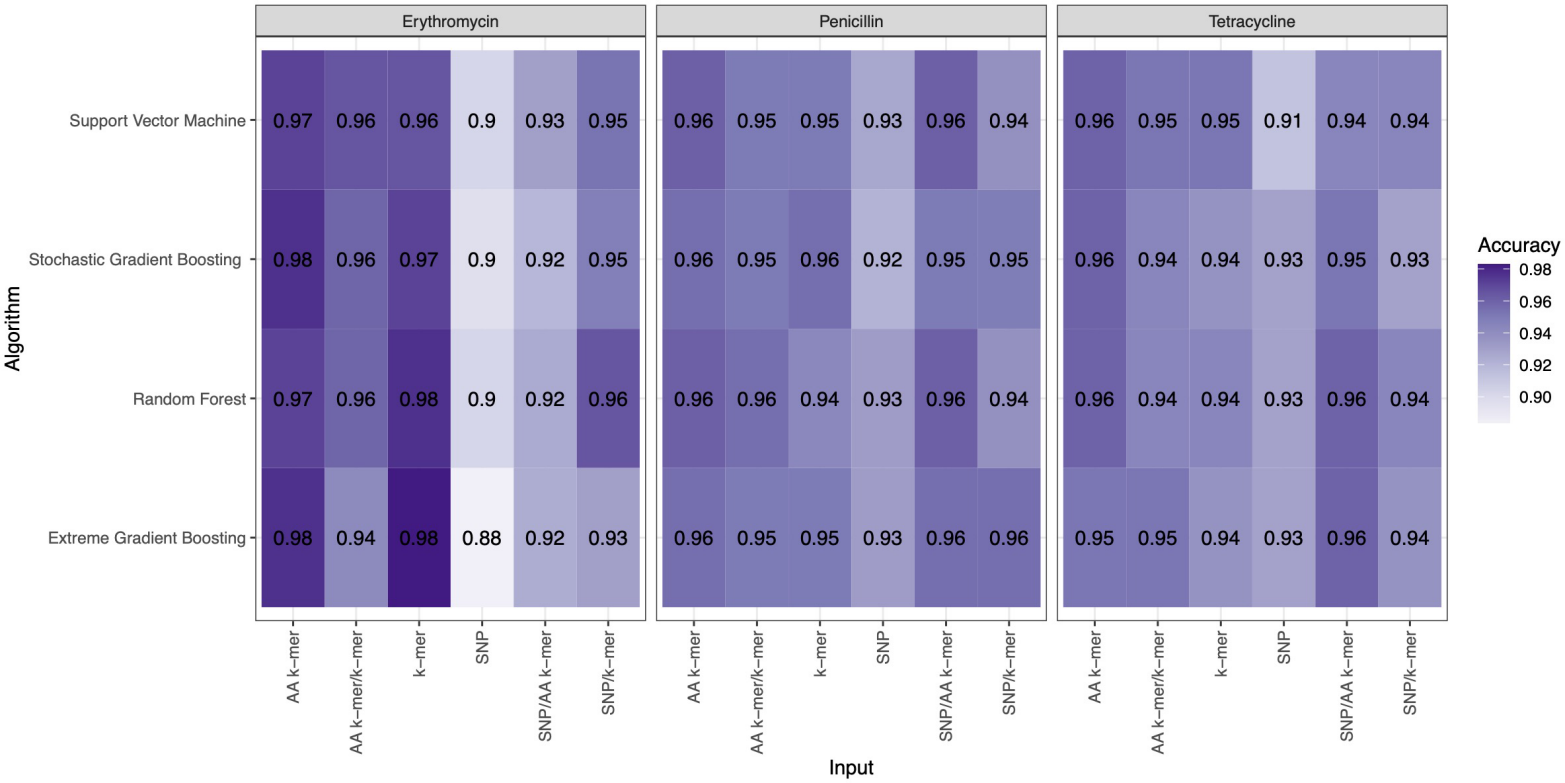
Accuracy results of penicillin, erythromycin, and tetracycline resistance classification models using six different input types and four different ML approaches.

**Figure 4:**
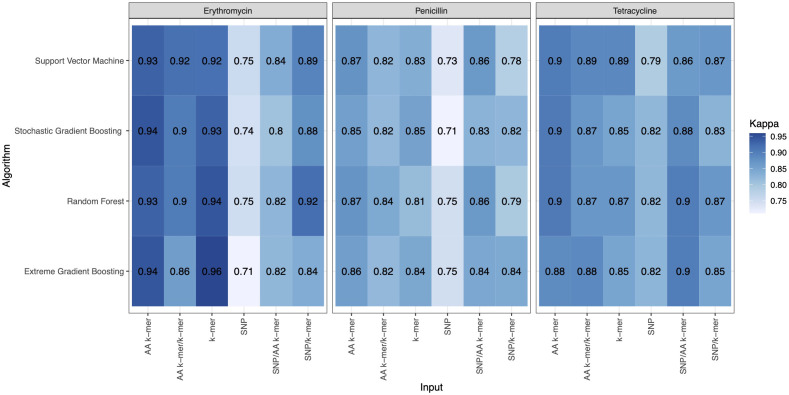
Kappa results of penicillin, erythromycin, and tetracycline resistance classification models using six different input types and four different ML approaches.

For penicillin, parameters were optimized *via* cross-validation, and performance estimates averaged over five repeats of this setup on 869 samples. For the prediction of penicillin susceptibility and resistance, the machine learning classifiers performed almost equally well with the five feature types (k-mer, AA k-mer, k-mer/AA k-mer, SNP/k-mer, and SNP/AA k-mer) except SNP alone itself. (all F1 score> 0.76). Comparisons of the models are shown in **Table 3** and **Figures 2**–**4**. These figures shows the F1 score, accuracy and kappa results of penicillin, erythromycin, and tetracycline resistance classification models using six different input types and four different ML approaches.

When different features for penicillin were compared, it was observed that nucleotide 10-mer and amino acid 5-mer F1 scores were higher than other features; amino acid 5-mer combinations were other inputs that yielded relatively higher F1 scores. SNP, SNP/AA k-mer, and SNP/k-mer combinations yielded lower accuracy and F1 scores than other penicillin models. The highest F1 score was observed in the Random Forest model at 0.9. The accuracy of the same model was found to be 0.96. The second-best option was SVM, and the third-best option was XGBoost, which performed almost as well as RF in F1-Score. The RF model utilizing 5-mer AA k-mer features to classify penicillin resistance yielded a sensitivity of 0.882 and a specificity of 0.99. Similarly, the SVM linear model resulted in high predictive sensitivity and specificity values of 0.86 and 0.99. Moreover, the XGBoost resulted in a sensitivity of 0.96 and a specificity of 0.93.

For erythromycin, a total of 895 samples were used in our models. As measured by the accuracy and F1 score, the best performance was achieved by nucleotide k-mer model with XGBoost (F1-Score 0.97, Accuracy 0.98). For the erythromycin AMR prediction, all classifiers performed almost equally well with all feature types except for SNP features. The first and second highest F1 score measured by XGBoost and random forest, which performed close to GBM in F1-Score and accuracy. When different features in erythromycin models were compared, it was seen for combinations of inputs, including SNP/AA k-mer, SNP/k-mer, and AA k-mer/k-mer, F1-scores were higher than SNP. Amino acid 5-mer and SNP combinations yielded the highest F1 scores. Performances of the erythromycin models are presented in **Table 4**. The second-best option was Random Forest in terms of F1-Score. With nucleotide k-mer feature, the erythromycin resistance classification using the XGBoost model correctly predicted resistance with a sensitivity of 0.87 and a specificity of 0.88.

Tetracycline parameters were optimized *via* cross-validation, and performance estimates were averaged over five repeats of this setup using 643 samples. Performances of all tetracycline models are presented in **Table 5**. For the prediction of tetracycline resistance, the ML classifiers performed almost equally well with the six input data types (k-mer, AA k-mer, SNPs, k-mer/AA k-mer, SNP/k-mer, and SNP/AA k-mer) (F1 score > 0.85, **Figure 2**). When different feature inputs in tetracycline were compared, it was observed that the AA k-mer/k-mer combination yielded a higher F1 score than other inputs. The highest F1 score was observed for the random forest model, with 0.93. The accuracy of the same model was found to be 0.96. The second-best option was GBM, and the third option was SVM, which performed close to RF in terms of F1-Score and accuracy. With the AA k-mer/k-mer feature tetracycline resistance RF model, tetracycline resistance could be predicted with a sensitivity of 0.85 and a specificity of 0.98.

As described in Methods, we used binary features (i.e., the presence or absence of k-mers) rather than k-mer counts to simplify the analyses. When a model is used to predict the MIC class for a new genome, the k-mers with the highest importance values are expected to be the most informative. Thus, by analyzing the feature importance values of each k-mer, we can use the models generated in this study to understand the genomic regions that differentiate MIC classes. Hence, to understand the relationship between known AMR genes and the important k-mers chosen by each model, we searched for k-mers with high-importance values within AMR genes or near an AMR gene.

In most cases, the top k-mers corresponded to known AMR genes. The top 10 10-mers with the highest feature importance values were checked against *S. pneumoniae*-related known AMR genes, including penicillin-binding proteins (PBPs), which have a major role in the cell wall synthesis (*PBP2b*, *PBP2x*, and *PBP1a*) and are most often associated with penicillin resistance. For macrolide resistance mechanisms in S. pneumoniae, *ermB* and *mefE* genes stand out, encoding an active efflux pump. Also, the most common resistance mechanism to tetracycline in S. pneumoniae is the acquisition of one of the three genes, *tetM*, *tetO*, and *tetK*. In the case of penicillin, for the top 10 features in *S. pneumoniae*, we used the hypergeometric test to calculate the probability of top 10 10-mers appearing in the resistance genes. As a result, p value (0.036) was found to be statistically significant when compared with pbp2b, pbp2x and pbp1a resistance genes. When we looked for tetracycline with tetS and tetM genes, the p value was 0.015. When we evaluated our 10-mers for Erythromycin, the rate of occurrence of nucleotide sequences in these genes for four genes (Ermb, mefE, msrD, mefA) was found to be 0.12.

## 4 Discussion

AMR has caused a significant increase in morbidity and mortality rate in infectious diseases worldwide, raising global health concerns (World Health Organization, 2019; World Health Organization, 2022a). It is crucial to quickly detect AMR in bacterial genomes as the number of effective antibiotics decreases. Molecular approaches have significantly improved over the years and play a critical role in the fight against antimicrobial resistance. (Leski et al., 2013; Inouye et al., 2014; Davis et al., 2016) Building classifiers with a balanced number of susceptible and resistant genomes is also important for building accurate classifiers but is currently a major limitation. In most cases, the number of available genomes with AMR data is resistant because these are of clinical importance to hospitals and epidemiologists.

Given the current data sets available on PATRIC, we built RF, SVM, GBM, and XGBoost classifiers for penicillin, erythromycin, and tetracycline resistance *Streptococcus pneumoniae*. The classifiers were highly accurate and performed classifications based on nucleotide k-mers.

The feature extraction methods that we present here have different pre-processing steps. In the case of SNP features, alignment to the reference genome is required because each SNP must have a unique position in the reference genome. In order to work with SNP locations, variant calling is required. Although it is not a very long process, it is a pre-process that should be evaluated. Apart from the location of the SNPs, we looked to see if there was a difference in the number of SNPs between susceptible and resistant samples, it was observed that the number of SNPs in the susceptible samples was significantly higher for all three antibiotics (**Figure S1**). The number of SNPs was significantly lower in the resistance samples regardless of antibiotic. This shows that simply assessing the number of SNPs in a sample might be a useful initial step when predicting MIC class.

When we compared the machine learning models, we could not find any obvious difference that could distinguish one from the other. When evaluating the results of Erythromycin, when we ran XGBoost, which gave F1 scores of 0.97 and 0.96, with the SNP feature, we saw that it gave the weakest result among the tested models (**Figures 2**–**4**). XGBoost is a popular machine learning algorithm it’s because of high predictive accuracy. XGBoost is fast and ideal for big datasets, when we compare to other models like random forest.

For amino acid k-mers methods, by contrast, the input to the feature extraction method is the amino acid sequence of the genes. This means that just aligning the short reads to contigs is not sufficient. This adds an extra pre-processing step to these methods. However, predicting AMR as fast as possible and as cheaply as possible is the top priority. Thus, amino acid k-mers are the better option because of the smaller feature size and better interpretability of AA features. Overall, aa kmer can be a useful tool for prediction, This method, which has just started to be used for MIC prediction, is seen to give high results when compared to other feature inputs.

Our comparisons showed that different feature inputs yielded the optimal results for each antibiotic. Amino acid 5-mers resulted in the best performance for penicillin. In contrast, the SNP and amino acid 5-mers combination were the best for tetracycline, and the combination of nucleotide 10-mers and amino acid 5-mers yielded the best performance for tetracycline.

In machine learning, an excessive number of features can increase the required memory and lead to over-fitting. Using long k-mers is hard because the number of features increases; however, we have shown that for amino acid k-mers, this increase in feature size is less severe than for nucleotide k-mers. One advantage of amino acid k-mers over nucleotide k-mers is that they are more compact representations of biological information. Each codon consists of three nucleotides and translates into one amino acid. Moreover, amino acid k-mers and their combinations achieved better performance in terms of accuracy.

In this study, the k-mers relating to penicillin resistance in *S. pneumoniae* that were identified by RF corresponded with the *pbp2x* gene that was also identified in previous genome-wide association studies (Chewapreecha et al., 2014). In that study, Chewapreecha and colleagues (2014) also found significant variations relating to resistance in the *pbp1a* and *pbp2a* penicillin-binding proteins, which were also identified in this study using the RF model.

In this study, we compared feature sets and ML models for predicting AMR phenotype for S. pneumoniae. We compared nucleotide k-mers, amino acid k-mers, and SNPs to predict AMR for three antibiotics: Penicillin, Erythromycin, and Tetracycline. Further, we attempted to use and compare various ML methods: random forest, support vector machine, stochastic gradient boosting, and extreme gradient boosting to train the classification models. We attempted to discuss the strengths and limitations of feature and ML model selection for MIC prediction. As a result of our work, we have observed that the features used in the model setup and the choice of ML method affect the result. Especially the feature combinations giving high accuracy and F1 score for some antibiotics showed that these feature inputs should be evaluated in the future. We hope that the approach undertaken by this study can be used in further studies to improve AMR prediction performance and accuracy and help alleviate the burden of AMR in the clinical setting.

## Data availability statement

The original contributions presented in the study are included in the article/**Supplementary Material**. Further inquiries can be directed to the corresponding author.

## Author contributions

First authorship: DK Senior authorship: EU, AK. Last authorship: OS. All authors contributed to the article and approved the submitted version.
