# Supplementary material for: A comparison of various feature extraction and machine learning methods for antimicrobial resistance prediction in streptococcus pneumoniae
Source: Front Antibiot. 2023 Mar 24;2:1126468. doi: 10.3389/frabi.2023.1126468 (PMC11731958; doi:10.3389/frabi.2023.1126468)
Supplement: Supplementary file 1 [file DataSheet_1.docx]

Supplementary Material

Comparison of Various Feature Extraction and Machine Learning Methods for Antimicrobial Resistance Prediction in *Streptococcus pneumoniae*

**Deniz Ece Kaya^1^, Ege Ülgen^1^, Ayşe Sesin Kocagöz^2^, Osman Uğur Sezerman^1^**

1: Department of Biostatistics and Medical Informatics, School of Medicine, Acibadem Mehmet Ali Aydinlar University, Istanbul, Turkey.

2: Department of Infectious Diseases, School of Medicine, Acibadem Mehmet Ali Aydinlar University, Istanbul, Turkey.

*** Correspondence:**Deniz Ece Kaya
[denizecek@gmail.com](mailto:denizecek@gmail.com)

**Supplementary Figure 1** Comparison of the distribution of the number of SNPs per MIC class per each antibiotic.

**Supplementary Table 2.** The best parameters selected for each antibiotic in the final models

| Algorithm | Antibiotic | input | Final Parameters |
| --- | --- | --- | --- |
| Random Forest | Penicillin | k-mer | mtry = 14 |
| Support Vector Machine | Penicillin | k-mer | sigma = 0.0063 and C = 16.54838 |
| Stochastic Gradient Boosting | Penicillin | k-mer | n.trees = 524, interaction.depth = 5, shrinkage = 0.1646977 and n.minobsinnode = 10 |
| Extreme Gradient Boosting | Penicillin | k-mer | nrounds = 100, max_depth = 5, eta = 0.3, gamma = 0, colsample_bytree =  1, min_child_weight = 1 and subsample = 1 |
| Random Forest | Penicillin | AA k-mer | mtry = 31 |
| Support Vector Machine | Penicillin | AA k-mer | degree = 3, scale = 0.63 and C = 0.61 |
| Stochastic Gradient Boosting | Penicillin | AA k-mer | n.trees = 850, interaction.depth = 3, shrinkage = 0.1 and n.minobsinnode = 10. |
| Extreme Gradient Boosting | Penicillin | AA k-mer | nrounds = 150, max_depth = 5, eta = 0.3, gamma = 0, colsample_bytree =  0.625, min_child_weight = 1 and subsample = 1 |
| Random Forest | Penicillin | SNP | mtry = 3 |
| Support Vector Machine | Penicillin | SNP | degree = 3, scale = 2.441974e-05 and C = 635.1844 |
| Stochastic Gradient Boosting | Penicillin | SNP | n.trees = 50, interaction.depth = 4, shrinkage = 0.1 and n.minobsinnode = 10. |
| Extreme Gradient Boosting | Penicillin | SNP | nrounds = 429, max_depth = 8, eta = 0.3588676, gamma = 9.13, colsample_bytree =  0.46, min_child_weight = 2 and subsample = 0.77 |
| Random Forest | Penicillin | SNP/AA k-mer | mtry = 13.67479 |
| Support Vector Machine | Penicillin | SNP/AA k-mer | degree = 3, scale = 2.441974e-05 and C = 635.1844 |
| Stochastic Gradient Boosting | Penicillin | SNP/AA k-mer | n.trees = 150, interaction.depth = 3, shrinkage = 0.1 |
| Extreme Gradient Boosting | Penicillin | SNP/AA k-mer | nrounds = 100, max_depth = 1, eta = 0.4, gamma = 0, colsample_bytree = 0.8, min_child_weight = 1 and subsample = 0.75. |
| Random Forest | Penicillin | SNP/k-mer | mtry = 12.76715 |
| Support Vector Machine | Penicillin | SNP/k-mer | sigma = 0.0016 and C = 5.84. |
| Stochastic Gradient Boosting | Penicillin | SNP/k-mer | n.trees = 1150, interaction.depth = 2, shrinkage = 0.1 and n.minobsinnode = 10. |
| Extreme Gradient Boosting | Penicillin | SNP/k-mer | nrounds = 816, max_depth = 2, eta = 0.1279373, gamma = 8.320024, colsample_bytree = 0.3631166, min_child_weight = |
| Random Forest | Penicillin | AA k-mer/k-mer | mtry = 5 |
| Support Vector Machine | Penicillin | AA k-mer/k-mer | sigma = 0.001939746 and C = 0.2004302 |
| Stochastic Gradient Boosting | Penicillin | AA k-mer/k-mer | n.trees = 200, interaction.depth = 3, shrinkage = 0.1 and n.minobsinnode = 10. |
| Extreme Gradient Boosting | Penicillin | AA k-mer/k-mer | nrounds = 150, max_depth = 3, eta = 0.4, gamma = 0, colsample_bytree = 0.6, min_child_weight = 1 and subsample = 0.5 |
| Random Forest | Erythromycin | k-mer | mtry = 20 |
| Support Vector Machine | Erythromycin | k-mer | sigma = 0.00904821 and C = 3.07926 |
| Stochastic Gradient Boosting | Erythromycin | k-mer | n.trees = 250, interaction.depth = 4, shrinkage = 0.1 and n.minobsinnode = 10 |
| Extreme Gradient Boosting | Erythromycin | k-mer | nrounds = 100, max_depth = 5, eta = 0.3, gamma = 0, colsample_bytree = 0.5, min_child_weight = 1 |
| Random Forest | Erythromycin | AA k-mer | mtry = 12.80 |
| Support Vector Machine | Erythromycin | AA k-mer | C = 0.6315789 |
| Stochastic Gradient Boosting | Erythromycin | AA k-mer | n.trees = 500, interaction.depth = 3, shrinkage = 0.1 and n.minobsinnode = 10 |
| Extreme Gradient Boosting | Erythromycin | AA k-mer | nrounds = 100, max_depth = 3, eta = 0.3, gamma = 0, colsample_bytree = 0.8, min_child_weight = 1# and subsample = 0.5. |
| Random Forest | Erythromycin | SNP | mtry = 12 |
| Support Vector Machine | Erythromycin | SNP | C = 0.73 |
| Stochastic Gradient Boosting | Erythromycin | SNP | n.trees = 500, interaction.depth = 3, shrinkage = 0.1 and n.minobsinnode = 10 |
| Extreme Gradient Boosting | Erythromycin | SNP | nrounds = 150, max_depth = 3, eta = 0.4, gamma = 0, colsample_bytree = 0.675, min_child_weight = 1 subsample = 0.5 |
| Random Forest | Erythromycin | SNP/AA k-mer | mtry = 30 |
| Support Vector Machine | Erythromycin | SNP/AA k-mer | sigma = 0.00035 and C = 211.443 |
| Stochastic Gradient Boosting | Erythromycin | SNP/AA k-mer | n.trees = 350, interaction.depth = 3, shrinkage = 0.1 and n.minobsinnode = 10 |
| Extreme Gradient Boosting | Erythromycin | SNP/AA k-mer | nrounds = 200, max_depth = 2, eta = 0.3, gamma = 0, colsample_bytree = 0.4, min_child_weight = 2 and subsample = 1 |
| Random Forest | Erythromycin | SNP/k-mer | mtry = 25 |
| Support Vector Machine | Erythromycin | SNP/k-mer | sigma = 0.01002054 and C = 10.98855 |
| Stochastic Gradient Boosting | Erythromycin | SNP/k-mer | n.trees = 150, interaction.depth = 3, shrinkage = 0.1 and n.minobsinnode = 10 |
| Extreme Gradient Boosting | Erythromycin | SNP/k-mer | nrounds = 100, max_depth = 3, eta = 0.3, gamma = 0, colsample_bytree = 0.3, min_child_weight = 1and subsample = 1 |
| Random Forest | Erythromycin | AA k-mer/k-mer | mtry = 45 |
| Support Vector Machine | Erythromycin | AA k-mer/k-mer | degree = 2, scale = 0.009069009 and C = 1.241622 |
| Stochastic Gradient Boosting | Erythromycin | AA k-mer/k-mer | n.trees = 3650, interaction.depth = 9, shrinkage = 0.01502375 and n.minobsinnode = 5 |
| Extreme Gradient Boosting | Erythromycin | AA k-mer/k-mer | nrounds = 200, max_depth = 3, eta = 0.3, gamma = 0, colsample_bytree = 0.3, min_child_weight = 2 and subsample = 1 |
| Random Forest | Tetracycline | k-mer | mtry = 7.61 |
| Support Vector Machine | Tetracycline | k-mer | sigma = 0.06456923 and C = 0.08250606 |
| Stochastic Gradient Boosting | Tetracycline | k-mer | n.trees = 150, interaction.depth = 2, shrinkage = 0.1 and n.minobsinnode = 10 |
| Extreme Gradient Boosting | Tetracycline | k-mer | nrounds = 150, max_depth = 1, eta = 0.4, gamma = 0, colsample_bytree = 0.8, min_child_weight = 1 |
| Random Forest | Tetracycline | AA k-mer | mtry = 11.40 |
| Support Vector Machine | Tetracycline | AA k-mer | sigma = 0.1153326 and C = 28.17864 |
| Stochastic Gradient Boosting | Tetracycline | AA k-mer | n.trees = 500, interaction.depth = 3, shrinkage = 0.1 and n.minobsinnode = 10 |
| Extreme Gradient Boosting | Tetracycline | AA k-mer | nrounds = 50, max_depth = 3, eta = 0.3, gamma = 0, colsample_bytree = 0.4, min_child_weight = 3 and subsample = 1 |
| Random Forest | Tetracycline | SNP | mtry = 14 |
| Support Vector Machine | Tetracycline | SNP | sigma = 0.002651096 and C = 210.9548 |
| Stochastic Gradient Boosting | Tetracycline | SNP | n.trees = 300, interaction.depth = 3, shrinkage = 0.1 and n.minobsinnode = 10 |
| Extreme Gradient Boosting | Tetracycline | SNP | nrounds = 150, max_depth = 3, eta = 0.3, gamma = 0, colsample_bytree = 0.6, min_child_weight = 1 subsample = 0.75. |
| Random Forest | Tetracycline | SNP/AA k-mer | mtry = 47 |
| Support Vector Machine | Tetracycline | SNP/AA k-mer | sigma = 0.0005349413 and C = 522.9208 |
| Stochastic Gradient Boosting | Tetracycline | SNP/AA k-mer | n.trees = 696, interaction.depth = 2, shrinkage = 0.04072671 and n.minobsinnode = 21 |
| Extreme Gradient Boosting | Tetracycline | SNP/AA k-mer | nrounds = 200, max_depth = 2, eta = 0.3, gamma = 0, colsample_bytree = 0.3, min_child_weight = 1 and subsample = 1 |
| Random Forest | Tetracycline | SNP/k-mer | mtry = 31 |
| Support Vector Machine | Tetracycline | SNP/k-mer | C = 0.1052632 |
| Stochastic Gradient Boosting | Tetracycline | SNP/k-mer | n.trees = 100, interaction.depth = 3, shrinkage = 0.1 and n.minobsinnode = 10 |
| Extreme Gradient Boosting | Tetracycline | SNP/k-mer | nrounds = 150, max_depth = 3, eta = 0.3, gamma = 0, colsample_bytree = 0.5, min_child_weight = 1 subsample = 0.5 |
| Random Forest | Tetracycline | AA k-mer/k-mer | mtry = 13.71 |
| Support Vector Machine | Tetracycline | AA k-mer/k-mer | sigma = 0.0008980129 and C = 336.0581 |
| Stochastic Gradient Boosting | Tetracycline | AA k-mer/k-mer | n.trees = 150, interaction.depth = 3, shrinkage = 0.1 and n.minobsinnode = 10 |
| Extreme Gradient Boosting | Tetracycline | AA k-mer/k-mer | nrounds = 150, max_depth = 3, eta = 0.3, gamma = 0, colsample_bytree = 0.4, min_child_weight = 2 subsample = 0.75 |

**Supplementary Table 3 :** Training and validation sets accuracy and kappa results of Penicillin

| Algorithm | Antibiotic | input | Training Accuracy | Training Kappa | Validation Accuracy | Validation Kappa |
| --- | --- | --- | --- | --- | --- | --- |
| Random Forest | Penicillin | k-mer | 0.965 | 0.884 | 0.943 | 0.813 |
| Support Vector Machine | Penicillin | k-mer | 0.970 | 0.902 | 0.926 | 0.776 |
| Stochastic Gradient Boosting | Penicillin | k-mer | 0.963 | 0.880 | 0.949 | 0.834 |
| Extreme Gradient Boosting | Penicillin | k-mer | 0.961 | 0.874 | 0.949 | 0.830 |
| Random Forest | Penicillin | AA k-mer | 0.960 | 0.864 | 0.941 | 0.801 |
| Support Vector Machine | Penicillin | AA k-mer | 0.941 | 0.801 | 0.958 | 0.855 |
| Stochastic Gradient Boosting | Penicillin | AA k-mer | 0.957 | 0.857 | 0.949 | 0.838 |
| Extreme Gradient Boosting | Penicillin | AA k-mer | 0.957 | 0.850 | 0.937 | 0.806 |
| Random Forest | Penicillin | SNP | 0.919 | 0.706 | 0.919 | 0.706 |
| Support Vector Machine | Penicillin | SNP | 0.917 | 0.699 | 0.913 | 0.675 |
| Stochastic Gradient Boosting | Penicillin | SNP | 0.913 | 0.682 | 0.909 | 0.664 |
| Extreme Gradient Boosting | Penicillin | SNP | 0.909 | 0.654 | 0.908 | 0.657 |
| Random Forest | Penicillin | SNP/AA k-mer | 0.965 | 0.883 | 0.965 | 0.882 |
| Support Vector Machine | Penicillin | SNP/AA k-mer | 0.968 | 0.890 | 0.966 | 0.890 |
| Stochastic Gradient Boosting | Penicillin | SNP/AA k-mer | 0.966 | 0.883 | 0.968 | 0.891 |
| Extreme Gradient Boosting | Penicillin | SNP/AA k-mer | 0.963 | 0.873 | 0.947 | 0.809 |
| Random Forest | Penicillin | SNP/k-mer | 0.971 | 0.903 | 0.971 | 0.903 |
| Support Vector Machine | Penicillin | SNP/k-mer | 0.975 | 0.916 | 0.968 | 0.890 |
| Stochastic Gradient Boosting | Penicillin | SNP/k-mer | 0.975 | 0.919 | 0.975 | 0.919 |
| Extreme Gradient Boosting | Penicillin | SNP/k-mer | 0.968 | 0.893 | 0.973 | 0.911 |
| Random Forest | Penicillin | AA k-mer/k-mer | 0.972 | 0.905 | 0.970 | 0.901 |
| Support Vector Machine | Penicillin | AA k-mer/k-mer | 0.972 | 0.906 | 0.951 | 0.845 |
| Stochastic Gradient Boosting | Penicillin | AA k-mer/k-mer | 0.971 | 0.902 | 0.958 | 0.854 |
| Extreme Gradient Boosting | Penicillin | AA k-mer/k-mer | 0.969 | 0.895 | 0.965 | 0.879 |

**Supplementary Table 4:** Training and validation sets accuracy and kappa results of Erythromycin

| Algorithm | Antibiotic | input | Training Accuracy | Training Kappa | Validation Accuracy | Validation Kappa |
| --- | --- | --- | --- | --- | --- | --- |
| Random Forest | Erythromycin | k-mer | 0.954 | 0.892 | 0.953 | 0.890 |
| Support Vector Machine | Erythromycin | k-mer | 0.946 | 0.874 | 0.907 | 0.776 |
| Stochastic Gradient Boosting | Erythromycin | k-mer | 0.941 | 0.861 | 0.941 | 0.861 |
| Extreme Gradient Boosting | Erythromycin | k-mer | 0.927 | 0.826 | 0.940 | 0.858 |
| Random Forest | Erythromycin | AA k-mer | 0.944 | 0.870 | 0.944 | 0.869 |
| Support Vector Machine | Erythromycin | AA k-mer | 0.951 | 0.887 | 0.939 | 0.857 |
| Stochastic Gradient Boosting | Erythromycin | AA k-mer | 0.947 | 0.876 | 0.946 | 0.875 |
| Extreme Gradient Boosting | Erythromycin | AA k-mer | 0.950 | 0.884 | 0.930 | 0.835 |
| Random Forest | Erythromycin | SNP | 0.872 | 0.668 | 0.879 | 0.695 |
| Support Vector Machine | Erythromycin | SNP | 0.881 | 0.705 | 0.857 | 0.638 |
| Stochastic Gradient Boosting | Erythromycin | SNP | 0.868 | 0.667 | 0.868 | 0.666 |
| Extreme Gradient Boosting | Erythromycin | SNP | 0.853 | 0.620 | 0.870 | 0.674 |
| Random Forest | Erythromycin | SNP/AA k-mer | 0.956 | 0.896 | 0.956 | 0.896 |
| Support Vector Machine | Erythromycin | SNP/AA k-mer | 0.950 | 0.884 | 0.916 | 0.797 |
| Stochastic Gradient Boosting | Erythromycin | SNP/AA k-mer | 0.944 | 0.866 | 0.940 | 0.857 |
| Extreme Gradient Boosting | Erythromycin | SNP/AA k-mer | 0.947 | 0.875 | 0.940 | 0.854 |
| Random Forest | Erythromycin | SNP/k-mer | 0.959 | 0.903 | 0.958 | 0.901 |
| Support Vector Machine | Erythromycin | SNP/k-mer | 0.946 | 0.871 | 0.957 | 0.899 |
| Stochastic Gradient Boosting | Erythromycin | SNP/k-mer | 0.958 | 0.902 | 0.958 | 0.902 |
| Extreme Gradient Boosting | Erythromycin | SNP/k-mer | 0.954 | 0.892 | 0.953 | 0.889 |
| Random Forest | Erythromycin | AA k-mer/k-mer | 0.960 | 0.908 | 0.954 | 0.894 |
| Support Vector Machine | Erythromycin | AA k-mer/k-mer | 0.956 | 0.898 | 0.934 | 0.840 |
| Stochastic Gradient Boosting | Erythromycin | AA k-mer/k-mer | 0.946 | 0.873 | 0.938 | 0.854 |
| Extreme Gradient Boosting | Erythromycin | AA k-mer/k-mer | 0.947 | 0.875 | 0.940 | 0.857 |

**Supplementary Table 5:** Training and validation sets accuracy and kappa results of Tetracycline

| Algorithm | Antibiotic | input | Training Accuracy | Training Kappa | Validation Accuracy | Validation Kappa |
| --- | --- | --- | --- | --- | --- | --- |
| Random Forest | Tetracycline | k-mer | 0.970 | 0.929 | 0.969 | 0.926 |
| Support Vector Machine | Tetracycline | k-mer | 0.971 | 0.931 | 0.971 | 0.931 |
| Stochastic Gradient Boosting | Tetracycline | k-mer | 0.961 | 0.906 | 0.963 | 0.912 |
| Extreme Gradient Boosting | Tetracycline | k-mer | 0.957 | 0.896 | 0.946 | 0.868 |
| Random Forest | Tetracycline | AA k-mer | 0.958 | 0.899 | 0.959 | 0.902 |
| Support Vector Machine | Tetracycline | AA k-mer | 0.952 | 0.882 | 0.961 | 0.906 |
| Stochastic Gradient Boosting | Tetracycline | AA k-mer | 0.956 | 0.895 | 0.958 | 0.899 |
| Extreme Gradient Boosting | Tetracycline | AA k-mer | 0.958 | 0.899 | 0.961 | 0.906 |
| Random Forest | Tetracycline | SNP | 0.932 | 0.829 | 0.936 | 0.840 |
| Support Vector Machine | Tetracycline | SNP | 0.903 | 0.763 | 0.903 | 0.763 |
| Stochastic Gradient Boosting | Tetracycline | SNP | 0.903 | 0.763 | 0.930 | 0.823 |
| Extreme Gradient Boosting | Tetracycline | SNP | 0.925 | 0.811 | 0.933 | 0.835 |
| Random Forest | Tetracycline | SNP/AA k-mer | 0.962 | 0.911 | 0.962 | 0.909 |
| Support Vector Machine | Tetracycline | SNP/AA k-mer | 0.954 | 0.889 | 0.938 | 0.849 |
| Stochastic Gradient Boosting | Tetracycline | SNP/AA k-mer | 0.955 | 0.890 | 0.948 | 0.872 |
| Extreme Gradient Boosting | Tetracycline | SNP/AA k-mer | 0.955 | 0.891 | 0.952 | 0.884 |
| Random Forest | Tetracycline | SNP/k-mer | 0.968 | 0.924 | 0.971 | 0.930 |
| Support Vector Machine | Tetracycline | SNP/k-mer | 0.971 | 0.930 | 0.963 | 0.910 |
| Stochastic Gradient Boosting | Tetracycline | SNP/k-mer | 0.955 | 0.892 | 0.957 | 0.896 |
| Extreme Gradient Boosting | Tetracycline | SNP/k-mer | 0.966 | 0.919 | 0.955 | 0.891 |
| Random Forest | Tetracycline | AA k-mer/k-mer | 0.970 | 0.927 | 0.970 | 0.927 |
| Support Vector Machine | Tetracycline | AA k-mer/k-mer | 0.965 | 0.916 | 0.963 | 0.911 |
| Stochastic Gradient Boosting | Tetracycline | AA k-mer/k-mer | 0.952 | 0.877 | 0.959 | 0.902 |
| Extreme Gradient Boosting | Tetracycline | AA k-mer/k-mer | 0.964 | 0.912 | 0.961 | 0.906 |
